# The Lower Limit of Reference of Urinary Albumin/Creatinine Ratio and the Risk of Chronic Kidney Disease Progression in Patients With Type 2 Diabetes Mellitus

**DOI:** 10.3389/fendo.2022.858267

**Published:** 2022-06-02

**Authors:** Wei-Hua Tang, Wei-Chin Hung, Chao-Ping Wang, Cheng-Ching Wu, Chin-Feng Hsuan, Teng-Hung Yu, Chia-Chang Hsu, Ya-Ai Cheng, Fu-Mei Chung, Yau-Jiunn Lee, Yung-Chuan Lu

**Affiliations:** ^1^ Division of Cardiology, Department of Internal Medicine, Taipei Veterans General Hospital, Yuli Branch, Hualien, Taiwan; ^2^ Faculty of Medicine, School of Medicine, National Yang Ming Chiao Tung University, Taipei, Taiwan; ^3^ Division of Cardiology, Department of Internal Medicine, E-Da Hospital, Kaohsiung, Taiwan; ^4^ School of Medicine, College of Medicine, I-Shou University, Kaohsiung, Taiwan; ^5^ School of Medicine for International Students, College of Medicine, I-Shou University, Kaohsiung, Taiwan; ^6^ Division of Cardiology, Department of Internal Medicine, E-Da Dachang Hospital, Kaohsiung, Taiwan; ^7^ Division of Gastroenterology and Hepatology, Department of Internal Medicine, E-Da Hospital, Kaohsiung, Taiwan; ^8^ The School of Chinese Medicine for Post Baccalaureate, College of Medicine, I-Shou University, Kaohsiung, Taiwan; ^9^ Department of Health Care Administration, College of Medicine, I-Shou University, Kaohsiung, Taiwan; ^10^ Lee’s Endocrinologic Clinic, Pingtung, Taiwan; ^11^ Division of Endocrinology and Metabolism, Department of Internal Medicine, E-Da Hospital, Kaohsiung, Taiwan

**Keywords:** Type 2 diabetes mellitus, low-grade albuminuria, risk, chronic kidney disease, progression

## Abstract

A urine albumin/creatinine ratio (UACR) <30 mg/g is considered to be normal, while increased risk of incident hypertension and cardiovascular disease mortality in subjects with high normal UACR level had been observed. However, a mild elevated but normal UACR level was associated with the risk of initiating chronic kidney disease (CKD) is uncertain. We investigated whether higher normal UACR is associated with the risk of developing CKD. A total of 4821 subjects with type 2 diabetes mellitus (T2DM), an estimated glomerular filtration rate >60 ml/min/1.73 m^2^ and UACR <30 mg/g enrolled in a diabetes disease management program between 2006 and 2020 were studied. The optimal cutoff point for baseline UACR as a predictor for progression to CKD according to the 2012 KDIGO definition was calculated using receiving operating characteristic curve analysis. After a mean of 4.9 years follow-up, the CKD risk progression increased in parallel with the quartiles of baseline UACR <30 mg/g (p for trend <0.0001). UACR cutoff points of 8.44 mg/g overall, 10.59 mg/g in males and 8.15 mg/g in females were associated with the risk of CKD progression. In multivariate Cox regression analysis, the hazard ratios for the association between UACR (>8.44 mg/g, >10.9 mg/g, >8.15 mg/g in overall, male, and female patients, respectively) and the risk of CKD progression were significant. This study demonstrated that a cutoff UACR value of >10 mg/g could significantly predict the cumulative incidence and progression of CKD in patients with T2DM.

## Introduction

Diabetes mellitus is currently the leading cause of chronic kidney disease (CKD) (1). Approximately 40% of patients with diabetes develop CKD, resulting in albuminuria, a reduction in glomerular filtration rate (GFR), or both (2). The main features of CKD are albuminuria and GFR <60 mL/min/1.73 m^2^, and they are well-known independent predictors of mortality, mainly from cardiovascular complications. A previous meta-analysis showed that the addition of urinary albumin-to-creatinine ratio (UACR) and GFR significantly improved the prediction of mortality and cardiovascular outcomes beyond traditional risk factors in patients with diabetes mellitus (3). Accordingly, the KDIGO 2012 CKD classification, which includes both GFR category and albuminuria stage, has been used to improve the accuracy of predicting the outcomes of CKD and cardiovascular disease (4).

A previous study demonstrated that moderately increased albuminuria was an indicator of subclinical organ damage, and that it may predict cardiovascular death independently of traditional risk estimators (systemic coronary risk evaluation) (5). Furthermore, moderately increased albuminuria, even below the normal limit of a UACR of 30 mg/g, has been reported to be an early diagnostic marker of diabetic nephropathy and kidney disease progression (6, 7). Before the estimated GFR (eGFR) falls to <60 ml/min/1.73 m^2^ and moderately increased albuminuria develops indicating kidney and vascular damage, there is a window below these values when early damage is already occurring.

In this regard, the cutoff values of normoalbuminuria and albuminuria are well established. If no other cardiovascular risk factors are present, clinical guidelines do not consider subjects with a UACR within the normal range (UACR <30 mg/g) to be at risk. However, clinical evidence has shown a continuous association between albuminuria levels and kidney function decline or cardiovascular disease, including at a low UACR of <10 mg/g (8, 9). Cardiorenal damage associated with albuminuria starts in normoalbuminuric conditions, and it has been associated with an increased incidence of cardiovascular events (10), subclinical atherosclerosis (11, 12) and heart failure (13) in the high-normal range. However, although it has been shown that low levels of albuminuria (i.e. UACR <30 mg/g) are associated with an increased risk of incident hypertension and cardiovascular mortality during follow-up (14), it is less clear whether mild elevated but normal UACR levels are associated with the risk of initiating CKD in patients with type 2 diabetes mellitus (T2DM). Therefore, we designed this study to investigate the relationship between the higher normal UACR and the risk of developing CKD in a T2DM cohort during a maximum of 14 years of follow-up.

## Materials and Methods

### Participants

In this longitudinal observational study, we included outpatients with T2DM who regularly attended eight diabetes specific clinics and the Diabetic Clinic of Kaohsiung E-Da Hospital, Taiwan. Patients were included if they were aged ≥18 years, had been clinically diagnosed with T2DM between January 2006 and October 2020, an eGFR ≥ 60 mL/min/1.73 m^2^, a UACR <30 mg/g, and no evidence suggesting the possibility of a non-diabetic renal disease. Non-diabetic renal diseases included drug-induced nephropathy, primary glomerular diseases, polycystic kidney, obesity-related nephropathy, reflux nephropathy, nephrolithiasis, and infectious diseases. The diagnosis of T2DM was based on World Health Organization criteria (15). In accordance with the diabetes comprehensive management program covered by the National Health Insurance system in Taiwan, patients were followed up at 3-month intervals. During the follow-up period, each patient underwent standardized physical examinations, biochemical measurements after fasting, and measurements of urine albumin and urine creatinine within a period of 3 months. All participants received treatment based on the standard strategies for diabetes, hypertension, and dyslipidemia during follow-up.

A total of 7,828 patients with T2DM who were managed in the comprehensive diabetes care program were collected. The exclusion criteria were an eGFR <60 mL/min/1.73 m^2^, a UACR ≥ 30 mg/g, patients with documented type 1 diabetes, patients with cancer, liver or urologic diseases, patients who were hospitalization within 3 months prior to enrollment or during the follow-up period, patients taking allopurinol or uricosuric agents for gouty arthritis, patients who underwent contrast examinations during the follow-up period, patients who could not provide complete information regarding demographics and medical history, and women who were pregnant. Also excluded from analysis were patients persistently showing hematuria and/or urinary casts to avoid the potential development/presence of primary glomerular diseases. Finally, 4,821 individuals were selected for the present analysis (**Supplementary Figure 1**). The study protocol and procedures were approved by the Ethics Committees of Pingtung Christian Hospital and E-Da Hospital with a Clinical Trial Approval Certificate from Pingtung Christian Hospital on 16th December 2005 and E-Da Hospital Institutional Review Board number EMRP-108-111. All experiments were carried out in accordance with the approved guidelines.

### Measurements

Routine tests performed during regular visits included a clinical examination, assessment of any possible adverse reactions and diet to prescribed medicines, body weight, blood pressure, urinary sediment and urinalysis using automated analyzers, complete blood count, serum chemistry, and HbA1c concentrations. The urinary albumin concentration was measured after overnight fasting by immunoturbidimetry (Beckman Instruments, Galway, Ireland). The detection limit was 2 mg/L, and the interassay and intraassay coefficients of variance were <8%. In the initial evaluation period, the patients (regardless of duration of diabetes) were defined as being normoalbuminuric if they had a UACR <30 mg/g in at least two consecutive overnight urine collections. In the follow-up period, to confirm the diagnosis of albuminuria, patients with a first UACR measurement >30 mg/g were asked to re-check the urine albuminuria level within 3 to 6 months. The CKD risk stage was defined according to eGFR and albuminuria categories following the KDIGO 2012 guidelines as: low risk (eGFR ≥ 60 mL/min/1.73 m^2^ and UACR <30 mg/g), moderately increased risk (eGFR >60 mL/min/1.73 m^2^ and 30<UACR<300 mg/g, or 45<eGFR<60 mL/min/1.73 m^2^ and 30<UACR<300 mg/g), high risk (30<eGFR<60 mL/min/1.73 m^2^ and UACR >300 mg/g, or eGFR >60 mL/min/1.73 m^2^ and UACR >300 mg/g), and very high risk (15<eGFR<60 mL/min/1.73 m^2^ and UACR >300 mg/g, or eGFR <15 mL/min/1.73 m^2^ and UACR >300 mg/g) (4). Each urine specimen was tested for the presence of urinary infections, and if present, the specimen was discarded and a new sample was collected after treatment. Normal serum creatinine levels (0.8-1.4 mg/dl) and normal urinary sediment (absence of protein, red blood cells, hemoglobin, white blood cells, nitrites and casts) were used to exclude primary renal diseases. Serum creatinine was measured using the Jaffe method. Renal function (eGFR) was calculated using the CKD-EPI two-concentration race equation (16): GFR = 141 × min(S_cr_/κ, 1)^α^ × max(S_cr/_κ, 1)^-1.209^ × 0.993^Age^ × 1.018 [if female] × 1.159 [if black], where S_cr_ is serum creatinine (mg/dL), κ is 0.7 for females and 0.9 for males, α is -0.329 for females and -0.411 for males, min indicates the minimum of S_cr_/κ or 1, and max indicates the maximum of S_cr_/κ or 1. In addition, hypertension was defined as a systolic blood pressure (SBP) ≥140mmHg, a diastolic blood pressure (DBP) ≥90mmHg, or if the patient was under antihypertensive treatment. Hyperlipidemia was defined as triglycerides ≥150 mg/dl, and/or high-density lipoprotein cholesterol (HDL-C) <35 mg/dl for men or <39 mg/dl for women, and/or total cholesterol ≥200 mg/dl, and/or low-density lipoprotein cholesterol (LDL-C) ≥130 mg/dl, or those undergoing treatment for lipid disorder according to the criteria of Adult Treatment Panel III.

Plasma biochemical parameters and urinary albumin were measured after an overnight fast. Serum HbA1C, total cholesterol, HDL-C, LDL-C, triglycerides, hemoglobin, creatinine, and glucose were determined by standard commercial methods on a parallel-multichannel analyzer (Hitachi 7170A, Tokyo, Japan) as in our previous reports (17, 18). Anthropometric parameters including body mass index (BMI) were measured. Seated blood pressure was measured by a trained nurse with a digital automatic blood pressure monitor (model HEM-907; Omron, Omron, Japan) after the subjects had rested for 5 minutes.

### Statistical Analysis

Data normality was analyzed using the Kolmogorov-Smirnov test. Continuous, normally distributed variables are presented as mean ± SD, and non-normally distributed variables as median (interquartile range). Categorical variables are presented as frequencies and/or percentages. All statistical analyses were performed using the Statistical Package for Social Science program (SPSS for Windows, version 12.0; SPSS, Chicago, IL). Clinical parameters and CKD risk stratified by UACR quartiles at baseline was tested for trends. Cox proportional hazards models were used to estimate hazard ratios (HRs) and 95% CIs for CKD risk progression in each quartile, compared with the lowest quartile as reference for urine UACR. For testing linear risk trends, we used the quartile rank as a continuous variable in the regression models. The optimal cutoff point of baseline UACR as a predictor for the initiation of advanced CKD (i.e., moderate risk to very high risk) was calculated using receiving operating characteristic (ROC) curve analysis. Hazard ratios (HRs) from Cox regression analysis were used to examine the variables that could predict the risk of advanced CKD in all patients, and then stratified by sex. We also investigated the risk of progression to advanced CKD (i.e., moderate risk to very high risk) in the patients who had a low risk of CKD. We first analyzed the parameters potentially related to CKD progression in univariate analysis and then in two multivariate Cox regression models. Model 1 used multivariate Cox regression analysis to assess the relationships of each parameter with the risk of CKD progression. Model 2 used multivariate forward stepwise Cox regression analysis that included variables with a p-value <0.1 in model 1. The Kaplan-Meier method and log-rank test were used to compare UACR categories stratified by cutoff points. Simple and multiple linear stepwise regression analyses were used to examine the associations and independence between UACR and the values of other parameters. A p-value <0.05 was considered to indicate statistical significance.

## Results

### Characteristics of Participants

The baseline characteristics and clinical data of the 4,821 patients enrolled in this study are presented in **Supplementary Table 1**. The mean ± SD age of the patients was 55.2 ± 12.5 years, and the median (interquartile range) known duration of diabetes was 4 years (1-9 years). The prevalence rates of hypertension, hyperlipidemia, and smoking were 15.7%, 27.0%, and 27.6%, respectively. The mean eGFR in all patients was 89.1 ± 20.5 mL/min/1.73 m^2^, and the median (interquartile range) UACR in all patients was 8.9 mg/g (4.7-15.7 mg/g). In addition, baseline clinical and biochemical characteristics stratified by UACR quartiles at baseline revealed that age, male sex, hypertension, hyperlipidemia, BMI, SBP, DBP, total cholesterol, triglycerides, HDL-C, LDL-C, fasting glucose, and HbA1c levels were significantly associated with UACR quartiles (p for trend<0.05, **Supplementary Table 2**).

### UACR and CKD Risk Progression

We investigated associations between baseline UACR quartiles and CKD risk progression. **Table 1** shows the HRs for the associations between UACR quartiles and CKD risk progression. After a mean 4.9 years of follow-up, the progression to moderately risk and very high risk stage of CKD increased in parallel with the quartiles (UACR <4.65 mg/g, UACR 4.65-8.93 mg/g, UACR 8.93-15.67 mg/g, and UACR ≥15.67 mg/g) of baseline UACR below 30 mg/g. As can be seen for progression to moderately risk and very high risk stage of CKD with each increasing quartile of UACR, there was an increase in the HR for progression to moderately risk and very high risk stage of CKD relative to Quartile 1, HR=1; Quartile 2, HR=1.01; Quartile 3, HR=1.44; Quartile 4, HR=2.29, p-value for the trend across increasing quartiles p<0.0001.

**Table 1:** Hazard ratios (HRs) of chronic kidney disease risk progression according to baseline urinary albumin/creatinine ratio quartiles followed for a mean 4.9 ± 4.0 years (N=4821).

Chronic kidney disease risk stage^†^				
Variables	Moderate risk to very high risk	Low risk	HR (95%CI)	p-value
Baseline UACR				
Q1 (<4.65 mg/g)	132 (11.0%)	1074	1.00 (reference)	
Q2 (4.65-8.93 mg/g)	232 (19.3%)	973	1.01 (0.82-1.26)	0.906
Q3 (8.93-15.67 mg/g)	313 (26.0%)	892	1.44 (1.18-1.77)	0.0004
Q4 (≥15.67 mg/g)	492 (40.8%)	713	2.29 (1.89-2.78)	<0.0001
p for trend			<0.0001	

UACR, urinary albumin/creatinine ratio. ^†^CKD risk stage was defined according to the 2012 KDIGO definition (4). HR, hazard ratio; CI, confidence interval.

### ROC Curve Analysis

**Figure 1:**
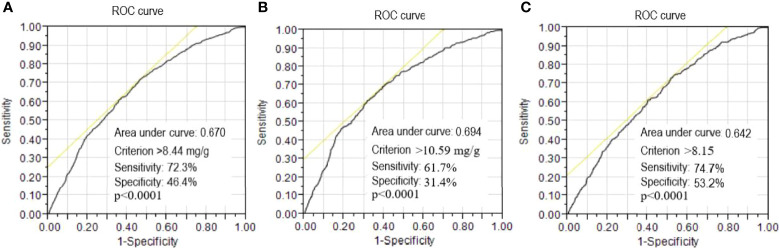
Receiver operating characteristic (ROC) curves of urinary albumin/creatinine ratio to detect the risk of progression to chronic kidney disease (CKD). A threshold value of >8.44 mg/g was associated with the risk of CKD progression with a sensitivity of 72.3% and specificity of 46.4% for all patients **(A)**. A threshold value of >10.59 mg/g was associated with the risk of CKD progression with a sensitivity of 61.7% and specificity of 31.4% for the male patients **(B)**. A threshold value of >8.15 mg/g was associated with the risk of CKD progression with a sensitivity of 74.7% and specificity of 53.2% for the female patients **(C)**.

ROC curve analysis to detect the risk of CKD progression revealed that the area under the curve (AUC) for baseline UACR was 0.670 (95% CI: 1.067-1.085, p<0.0001). A UACR cutoff point of 8.44 mg/g was associated with the risk of CKD progression, with a sensitivity of 72.3% and specificity of 46.4% for all patients (**Figure 1A**). Furthermore, in male patients, the AUC for baseline UACR was 0.694 (95% CI: 1.074-1.099, p<0.0001) to predict the risk of CKD progression. A UACR cutoff point of 10.59 mg/g was associated with the risk of CKD progression, with a sensitivity of 61.7% and specificity of 31.4% for the male patients (**Figure 1B**). In female patients, the AUC for baseline UACR was 0.642 (95% CI: 1.052-1.078, p<0.0001) to predict the risk of CKD progression. A UACR cut-off point of 8.15 mg/g was associated with the risk of CKD progression, with a sensitivity of 74.7% and specificity of 53.2% for the female patients (**Figure 1C**).

### Cox Regression Analysis UACR Cutoff Point and the Risk of CKD Progression in All Patients

In simple Cox regression analysis (**Table 2**), the baseline clinical and biochemical variables associated with the risk of CKD progression were age, eGFR, UACR >8.44 mg/g, BMI, SBP, DBP, total cholesterol, HDL-C, triglycerides, HbA1c, hemoglobin, statin treatment, and angiotensin converting enzyme inhibitor (ACEI) or angiotensin receptor blocker (ARB) treatment in all patients. In multivariate Cox regression analysis (**Table 2**), UACR >8.44 mg/g was confirmed to be an independent factor for the risk of CKD progression (HR=1.88, 95% CI, 1.65-2.14, p<0.0001) after adjustments for age, sex, disease duration, and baseline eGFR (model 1). In further multivariate stepwise Cox regression analysis (model 2), we found HDL-C level was negatively associated with the risk of CKD progression, and UACR >8.44 mg/g, smoking status, total cholesterol, HbA1c level, statin treatment, and ACEI/ARB treatment were positively related to the risk of CKD progression in all patients.

**Table 2:** Hazard ratios (HRs) of progression to moderately increased risk and very high risk stage of chronic kidney disease in type 2 diabetic patients with a low risk stage of chronic kidney disease.

	Univariate		Multivariate model 1		Multivariate model 2	
	HR (95% CI)	p-value	HR (95% CI)	p-value	HR (95% CI)	p-value
Age	1.03 (1.03-1.04)	<0.0001	–	–	–	–
Sex	0.99 (0.88-1.11)	0.816	–	–	–	–
Estimated glomerular filtration rate	0.99 (0.99-1.00)	<0.0001	–	–	–	–
UACR (>8.44 versus ≤8.44 mg/g)	1.89 (1.67-2.16)	<0.0001	1.88 (1.65-2.14)	<0.0001	1.81 (1.59-2.06)	<0.0001
Smoking (yes versus no)	1.04 (0.92-1.18)	0.544	1.21 (1.03-1.42)	0.018	1.03 (1.01-1.04)	0.0003
Body mass index (per unit)	1.04 (1.03-1.05)	<0.0001	1.06 (1.04-1.07)	<0.0001		
Systolic blood pressure (per unit)	1.01 (1.00-1.01)	<0.0001	1.00 (0.99-1.01)	0.146		
Diastolic blood pressure (per unit)	1.01 (1.00-1.01)	0.015	1.01(1.00-1.01)	0.002		
Total cholesterol (per unit)	1.00 (1.00-1.00)	0.008	1.00 (1.00-1.01)	<0.0001	1.00 (1.00-1.00)	0.014
HDL-cholesterol (per unit)	0.99 (0.99-1.00)	0.004	0.99 (0.99-1.00)	0.004	0.99 (0.99-1.00)	0.011
LDL-cholesterol (per unit)	1.00 (0.99-1.00)	0.117	1.00 (0.99-1.00)	0.437		
Triglycerides (per unit)	1.00 (1.00-1.00)	0.001	1.00 (1.00-1.00)	<0.0001		
HbA1c (per unit)	1.05 (1.02-1.08)	0.001	1.07 (1.04-1.10)	<0.0001	1.03 (1.00-1.06)	0.029
Hemoglobin (per unit)	0.94 (0.90-0.97)	0.001	0.97 (0.93-1.02)	0.220		
Statin treatment (yes versus no)	1.66 (1.42-1.93)	<0.0001	1.60 (1.36-1.86)	<0.0001	1.67 (1.43-1.94)	<0.0001
ACEI/ARB treatment (yes versus no)	1.84 (1.55-2.16)	<0.0001	1.66 (1.40-1.96)	<0.0001	1.58 (1.33-1.86)	<0.0001
Fibrate treatment (yes versus no)	1.35 (0.88-1.96)	0.160	1.68 (1.10-2.45)	0.019		

Multivariate model 1: Adjusted for age, sex, disease duration, and baseline estimated glomerular filtration rate. Multivariate model 2: multivariate stepwise. Cox regression analysis including all variables with a p-value <0.1 in model 1 listed in the table after adjustment for age, sex, disease duration, and baseline estimated glomerular filtration rate. UACR, urinary albumin/creatinine ratio; HDL-C, high-density lipoprotein cholesterol; LDL, low-density lipoprotein; ACEI/ARB, angiotensin converting enzyme inhibitor or angiotensin receptor blocker.

### UACR Cutoff Point and the Risk of CKD Progression in the Male Patients

When the patients were stratified by sex, simple Cox regression analysis showed that the baseline clinical and biochemical variables associated with the risk of CKD progression were UACR >10.59 mg/g, BMI, SBP, total cholesterol, HDL-C, triglycerides, HbA1c, hemoglobin, statin treatment, and ACEI/ARB treatment in the male patients (**Supplementary Table 3**). In multivariate Cox regression analysis (**Supplementary Table 3**), the association between UACR >10.59 mg/g and the risk of CKD progression (HR=2.35, 95% CI, 1.98-2.80, p<0.0001) remained statistically significant after adjustment for age, disease duration, and baseline eGFR (model 1). In further multivariate stepwise Cox regression analysis (model 2), while an appropriate HDL-C level was negatively associated with the risk of CKD progression, UACR >10.59 mg/g, SBP, total cholesterol, statin treatment, and ACEI/ARB treatment were positively related to the risk of CKD progression in the male patients.

### UACR Cutoff Point and the Risk of CKD Progression in the Female Patients

Furthermore, simple Cox regression analysis showed that the baseline clinical and biochemical variables associated with the risk of CKD progression were UACR >8.15 mg/g, BMI, SBP, DBP, HDL-C, triglycerides, HbA1c, hemoglobin, statin treatment, and ACEI/ARB treatment in the female patients (**Supplementary Table 4**). In multivariate Cox regression analysis (**Supplementary Table 4**), the association between UACR >8.15 mg/g and the risk of CKD progression (HR=1.46, 95% CI, 1.21-1.77, p<0.0001) remained statistically significant after adjustment for age, disease duration, and baseline eGFR (model 1). In further multivariate stepwise Cox regression analysis (model 2), while an appropriate hemoglobin level was negatively associated with the risk of CKD progression, UACR >8.15 mg/g, BMI, triglycerides, HbA1c, statin treatment, and ACEI/ARB treatment were positively related to the risk of CKD progression in female patients.

### UACR Was Associated With Cumulative Incidence of CKD Progression

**Figure 2:**
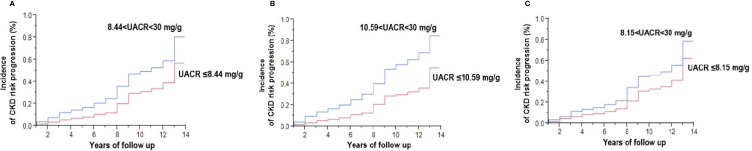
Kaplan-Meier analysis of the risk of progression to a moderately increased risk and very high risk stage of chronic kidney disease (CKD) in type 2 diabetic patients with a low risk stage of CKD **(A)**, and separately for male patients **(B)** and female patients **(C)**. UACR denotes urinary albumin-creatinine ratio expressed as mg albumin/g creatinine. Baseline UACR was defined as the mean of the first two consecutive measurements. See definition of moderately increased risk to very high-risk stage of CKD in the Materials and Methods.

In addition, Kaplan-Meier analysis showed that higher baseline UACR was associated with a significantly higher cumulative incidence of CKD progression in all patients (p<0.0001; **Figure 2A**), and separately for male patients (p<0.0001; **Figure 2B**) and female patients (p<0.0001; **Figure 2C**).

### Variables Associated With UACR Levels

Simple linear regression analysis revealed that UACR was positively associated with BMI, SBP, DBP, fasting glucose, HbA1c, total cholesterol, triglycerides, LDL-C, and uric acid. In addition, levels of HDL-C, eGFR, and hemoglobin were negatively associated with levels of UACR, while in multiple stepwise regression analysis, UACR level was positively associated with SBP, HbA1c, triglycerides and LDL-C, and negatively associated with HDL-C and hemoglobin (**Supplementary Table 5**).

## Discussion

In the present study, we demonstrated for the first time that in patients with diabetes and normal albuminuria, a lower limit of UACR (cutoff values of 8.44 mg/g overall, 10.59 mg/g in males and 8.15 mg/g in females, respectively) could be used to predict the risk of CKD progression and allow for intensive interventions. Renal damage and vascular dysfunction always occur eventually in patients with diabetes, causing a huge economic burden and patient disability (19–21). In the past decade, many major studies have focused on preventing the progression of renal damage in patients with diabetes and especially in those with a UACR >30 mg/g, however it is still not possible to prevent all such adverse events (22–25). Furthermore, in a recent study by Santiago-Hernandez et al. (26), early renal and vascular damage was shown in patients with normal albuminuria. Hence, in accordance with our findings, a UACR >10 mg/g was associated with the risk of CKD progression.

Among the 4,821 patients in our study population, the average known duration of diabetes was 4 years, and all traditional CKD risk factors were controlled, including blood pressure (average 129 ± 18 mmHg) and LDL (98.3 ± 29.9 mg/dl). Although the average HbA1c level was 8.4 ± 2.1%, which is higher than the target level of 7%, our study subjects represent a real-world heterogenous cohort of patients with diabetes. Interestingly, the females had a lower and more “sensitive” UACR cutoff value than the males. A similar phenomenon was also found in a previous study conducted in Korea and other epidemiological studies, in which a significant increase in the prevalence of CKD relative to the general population was found following the onset of menopause (27, 28). In the past decade, sex differences in CKD progression have been investigated, and CKD has been shown to affect more women than men, although the incidence of end-stage renal disease is higher in men than in women (27, 28). However, to date, the underlying mechanisms of how estrogen and testosterone contribute to the sex differences in the progression of CKD are still not fully understood. They are probably related to effects on modulating vascular tone, oxidative stress, inflammation, and apoptosis (29–31).

In multivariate Cox regression analysis in this study, we determined the HRs of known vascular damage risk factors for progression of CKD. None of the traditional risk factors such as smoking habit, blood pressure, total cholesterol, triglycerides or HbA1c showed significant effects on CKD progression, even though there were significant p values (**Table 2**, and **Supplementary Tables 3, 4**). This may be because all of the patients were enrolled in our diabetes disease management program and all of the modifiable risk factors were under reasonable control. Our analysis showed that the use of statins and renin-angiotensin- aldosterone system antagonists was related to the progression of CKD. This could be because the participants in our study already received sufficient care for metabolic syndrome and hypertension from the beginning of treatment. In addition, this may also explain why the traditional risk factors in our study did not affect the progression of CKD, and show the emerging importance of the role of UACR in the early stage of renal disease in diabetic patients.

In analysis of the female participants, a lower hemoglobin level was associated with progression of CKD (**Supplementary Table 4**). This also demonstrates that anemia, which can cause chronic renal hypoxia, is a non-conventional risk factor associated with CKD (32). The importance of controlling anemia has been reported to be important during and even before the progression of CKD and not only after end-stage renal disease in female diabetic patients (32, 33). The lack of a hemoglobin effect in the males in our analysis also demonstrates that there is a sex difference in CKD similar with many other cardiovascular diseases (33–35).

Although recent studies have reported that early renal and vascular damage occurs in normoalbuminuric conditions, we further showed a significant difference in CKD progression among different levels of UACR in diabetes patients with normal albuminuria. The progression of albuminuria differs in different populations, and the molecular mechanisms underlying the development of albuminuria and the biological variability between individuals in the early stages of CKD are still not fully understood. In recent study, diabetes patients were associated with high prevalence of non-alcoholic fatty liver disease (NAFLD), which was associated with a higher risk of impaired renal function (36). It is interested whether the NAFLD also take part in the early progression of renal impairment in diabetes patient. While, it is impossible to performed liver echography to evaluate all diabetes patient in real world practice that we could not adjust the effect of NAFLD on CKD development; as a result, focus is still placed on traditional CKD risk factors or coronary artery disease (CAD) risk management in these patients (37). It is therefore important to elucidate strategies to help prevent CKD progression in diabetic patients, including strategies to prevent increases in UACR. In our study, control of traditional risk factors such as blood pressure, HbA1c, LDL and anemia still played an important role in UACR progression (**Supplementary Table 5**), and further investigations are needed to elucidate whether stricter SBP and LDL targets for diabetic patients similar to those with CAD should be considered (38, 39). Nevertheless, our results showed that the cumulative incidence of CKD progression among the patients whose UACR level was lower than the cut cutoff value (8.44 mg/g, 10.59 mg/g, 8.15 mg/g, in overall, male, and female patients, respectively) was significantly lower compared to those who had a UACR level higher than the cutoff value (p<0.0001, **Figure 2**). Therefore, we suggest that in diabetic patients with normal albuminuria and a UACR greater than these cutoff values, more intensive LDL control, strict blood pressure control, active lifestyle modifications, detection of anemia and treatment should be applied. In the other hand, an increased UACR within the normal range has been proved independently associated with a higher risk of hypertension, and a systemic hypertension will alternate renal glomerular circulation and consequence to cause renal function impairment (40, 41). Whether early detection of renal hyperperfusion in type 2 diabetes might be a manifestation of normal urinary protein should be considered. Unfortunately, it is relative not easy to determine the early renal perfusion abnormality, further evaluation of the association of UACR levels and early abnormal renal perfusion status should be further investigated in the future (42, 43).

The main limitation in this study is that we only focused on diabetic patients with normal albuminuria. Whether our results can also be applied to non-diabetic patients with early CKD is unknown. In addition, previous studies have shown the prediabetes increased the risk of all-cause mortality, cardiovascular disease, and heart failure in the general population, especially in individuals with atherosclerotic cardiovascular disease (44, 45). Whether a mild elevated but normal UACR level could also predict the risk macrovascular and microvascular disease in prediabetes is worth to be explored. Second, there was a significant difference in the UACR cutoff values between the male and female patients. Further studies with a larger sample sized are needed to elucidate whether there is a more appropriate universal UACR cutoff value. Third, the underlying biochemical and biophysiological mechanisms underlying our observations should be investigated. Whether other clinical serum markers such as known inflammatory markers and uremic toxins are involved in CKD progression in patients with a “normal” UACR should also be clarified (17, 18, 46, 47).

In conclusion, a cutoff UACR value of >10 mg/g could significantly predict the cumulative incidence and progression of CKD in patients with T2DM. UACR measurements should be performed in all diabetic patients, even in those with normal albuminuria.

## Data Availability Statement

The raw data supporting the conclusions of this article will be made available by the authors, without undue reservation.

## Ethics Statement

The studies involving human participants were reviewed and approved by Pingtung Christian Hospital and E-Da Hospital. The patients/participants provided their written informed consent to participate in this study. Written informed consent was obtained from the individual(s) for the publication of any potentially identifiable images or data included in this article

## Author Contributions

All authors contributed to this study. W-HT and Y-CL conceived and designed the study. W-CH, Y-JL, and Y-CL provided the methodology. F-MC and Y-AC performed the formal analysis, and project administration. Y-CL and Y-JL validated the data. Y-JL, C-PW, T-HY, and Y-CL performed the investigation, resources, and data curation. W-HT, C-FH, C-CW, C-PW, and C-CH prepared the manuscript. W-HT, C-CW, W-CH, C-FH, C-CH, and T-HY reviewed and edited the manuscript. W-HT, Y-JL, and Y-CL performed the visualization. Y-CL performed the supervision and funding acquisition. All authors have read and agreed to the published version of the manuscript. All authors contributed to the article and approved the submitted version.

## Funding

This work was supported by grants from E-Da Hospital of the Republic of China, Taiwan (contract no. EDAHI109002 and EDAHI110001).

## Conflict of Interest

The authors declare that the research was conducted in the absence of any commercial or financial relationships that could be construed as a potential conflict of interest.

## Publisher’s Note

All claims expressed in this article are solely those of the authors and do not necessarily represent those of their affiliated organizations, or those of the publisher, the editors and the reviewers. Any product that may be evaluated in this article, or claim that may be made by its manufacturer, is not guaranteed or endorsed by the publisher.
