# Supplementary material for: The Lower Limit of Reference of Urinary Albumin/Creatinine Ratio and the Risk of Chronic Kidney Disease Progression in Patients With Type 2 Diabetes Mellitus
Source: Front Endocrinol (Lausanne). 2022 Jun 2;13:858267. doi: 10.3389/fendo.2022.858267 (PMC9200995; doi:10.3389/fendo.2022.858267)
Supplement: Supplementary file 1 [file DataSheet_1.pdf]

SUPPLEMENTARY DATA

The lower limit of reference of urinary albumin/creatinine ratio and the risk of chronic kidney disease progression in patients with type 2 diabetes mellitus

Contents

Supplementary Figure 1. Flow of participants in the comprehensive diabetes care……2

Supplementary Table 1. Baseline characteristics of the study population……………….3

Supplementary Table 2. Baseline clinical and biochemical characteristics stratified by urinary albumin/creatinine ratio quartiles at baseline ………………………………………4

Supplementary Table 3. Hazard ratios (HRs) of progression to moderately increased risk and very high risk stage of chronic kidney disease in male type 2 diabetic patients with a low risk stage of chronic kidney disease……………………………………………..5

Supplementary Table 4. Hazard ratios (HRs) of progression to moderately increased risk and very high risk stage of chronic kidney disease in female type 2 diabetic patients with a low risk stage of chronic kidney disease……………………………………………..6

Supplementary Table 5. Linear regression analysis of variables associated with urinary albumin/creatinine ratio levels………………………………………………………………7

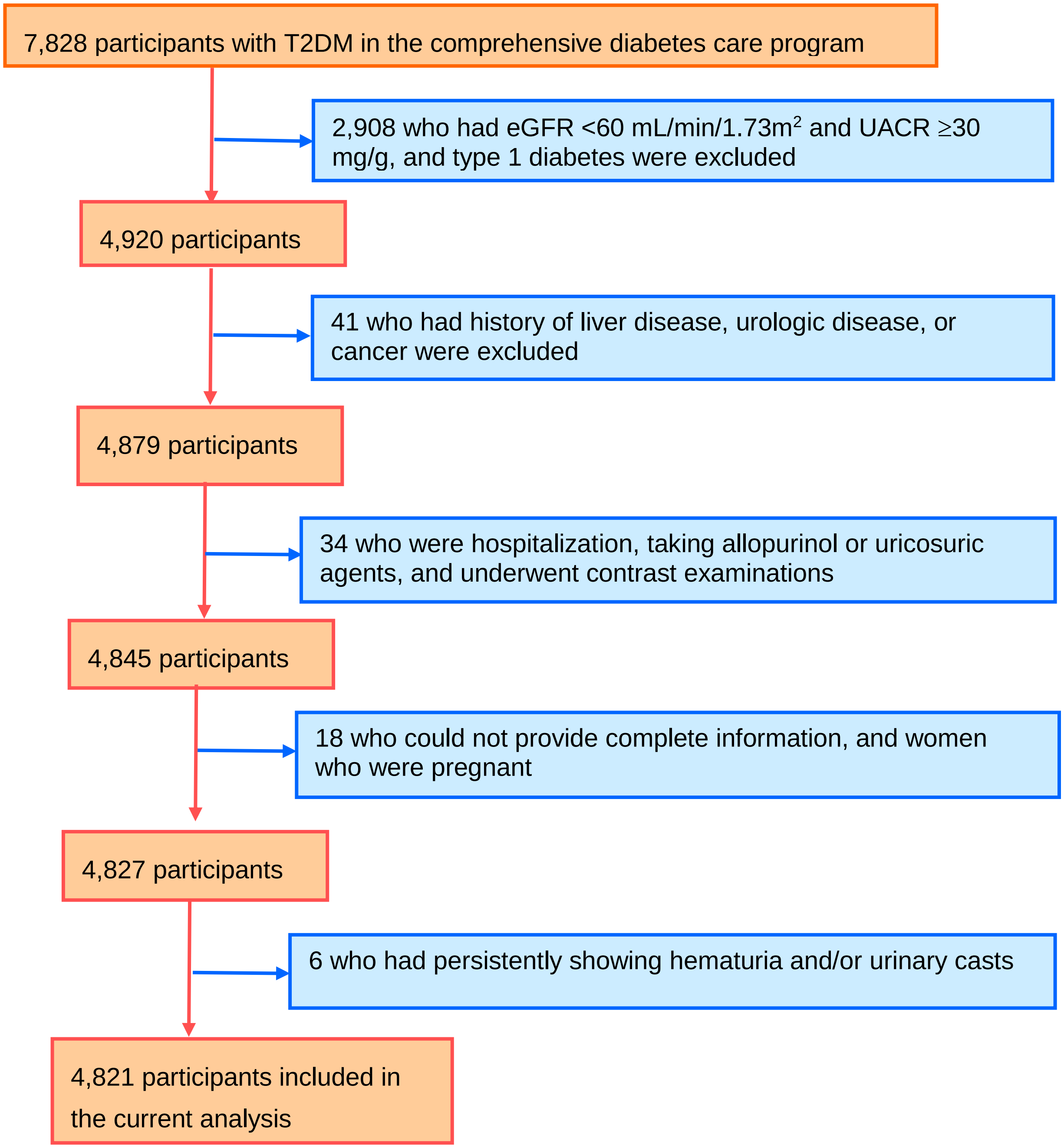

Supplementary Figure 1. Flow of participants in the comprehensive diabetes care

Supplementary Table 1. Baseline characteristics of the study population

Characteristic	Total (N = 4821)
Age (years)	55.2 ± 12.5
Male (n, %)	2,479 (51.4)
Known diabetes duration (years)	4 (1-9)
Hypertension (n, %)	757 (15.7)
Hyperlipidemia (n, %)	1301 (27.0)
Smoker (n, %)	1331 (27.6)
eGFR (ml/min/1.73 m2)	89.1 ± 20.5
Urinary albumin/creatinine ratio (mg/g)	8.9 (4.7-15.7)
Body mass index (kg/m^2)	26.1 ± 4.2
Systolic blood pressure (mmHg)	129 ± 18
Diastolic blood pressure (mmHg)	77 ± 12
Total cholesterol (mg/dl)	185.1 ± 37.6
Triglycerides (mg/dl)	116.0 (83.0-167.0)
HDL-cholesterol (mg/dl)	51.2 ± 13.5
LDL-cholesterol (mg/dl)	98.3 ± 29.9
Fasting glucose (mg/dl)	156.4 ± 57.1
HbA1c (%)	8.4 ± 2.1
Statins (n, %)	1197 (24.8)
Fibrate (n, %)	96 (2.0)
ACEI/ARB (n, %)	535 (11.1)

Data are expressed as the mean ± SD, number (percentage), or median (interquartile range). eGFR, estimated glomerular filtration rate; HDL, high-density lipoprotein; LDL, low-density lipoprotein; ACEI/ARB, angiotensin converting enzyme inhibitor or angiotensin receptor blocker.

Supplementary Table 2. Baseline clinical and biochemical characteristics stratified by urinary albumin/ creatinine ratio quartiles at baseline (N=4821)

Variable	Q1 (<4.65 mg/g)	Q2 (4.65-8.93 mg/g)	Q3 (8.93-15.67 mg/g)	Q4 (≥15.67 mg/g)	p for trend
No	1206	1205	1205	1205	
Age (years)	53.9±12.8	55.0±12.3	55.7±12.4	56.2±12.2	<0.0001
Male (n, %)	503(41.7)	558(46.3)	661(54.9)	620(51.5)	<0.0001
Hypertension (n, %)	161(13.4)	176(14.6)	188(15.6)	232(19.3)	<0.0001
Hyperlipidemia (n, %)	364(30.2)	339(28.1)	292(24.2)	306(25.4)	0.001
BMI (kg/m^2)	25.9±4.2	25.8±4.0	26.0±4.0	26.7±4.7	<0.0001
SBP (mmHg)	125±17	129±17	131±18	133±18	<0.0001
DBP (mmHg)	75±12	76±11	77±12	79±12	<0.0001
TCHOL (mg/dl)	181.8±37.8	185.1±36.9	185.9±37.6	187.6±38.1	0.0002
TGs (mg/dl)	106(78-150)	113(79-164)	116(85-167)	129(90-190)	<0.0001
HDL-C (mg/dl)	52.6±13.9	51.2±13.8	50.9±13.5	50.0±12.7	<0.0001
LDL-C (mg/dl)	96.0±30.1	99.3±29.5	99.0±30.0	99.1±30.1	0.020
Fasting glucose (mg/dl)	153.3±59.1	154.1±54.8	156.8±58.0	161.6±56.0	0.0002
HbA1c (%)	8.2±2.1	8.3±2.0	8.5±2.0	8.6±2.1	<0.0001

Data are expressed as the mean ± SD or median (interquartile range). BMI, body mass index; SBP, systolic blood pressure; DBP, diastolic blood pressure; TCHOL, total cholesterol; TGs, triglycerides; HDL, high-density lipoprotein-cholesterol; LDL-C, low-density lipoprotein-cholesterol.

Supplementary Table 3. Hazard ratios (HRs) of progression to moderately increased risk and very high risk stage of chronic kidney disease in male type 2 diabetic patients with a low risk stage of chronic kidney disease

	Univariate		Multivariate model 1		Multivariate model 2	
	HR (95% CI)	p-value	HR (95% CI)	p-value	HR (95% CI)	p-value
UACR (>10.59 versus ≤10.59 mg/g)	2.33 (1.97-2.76)	<0.0001	2.35 (1.98-2.80)	<0.0001	2.28 (1.93-2.71)	<0.0001
Smoking (yes versus no)	1.08 (0.92-1.28)	0.354	1.21 (1.02-1.43)	0.031		
Body mass index (per unit)	1.04 (1.02-1.06)	0.001	1.07 (1.04-1.09)	<0.0001		
Systolic blood pressure (per unit)	1.01 (1.00-1.01)	0.002	1.00 (0.99-1.01)	0.097	1.01 (1.00-1.02)	0.002
Diastolic blood pressure (per unit)	1.00 (0.99-1.01)	0.247	1.01 (0.99-1.02)	0.059		
Total cholesterol (per unit)	1.00 (1.00-1.01)	0.027	1.00 (1.00-1.01)	0.001	1.00 (1.00-1.01)	0.024
HDL-cholesterol (per unit)	0.99 (0.99-1.00)	0.027	0.99 (0.98-1.00)	0.013	0.99 (0.98-0.99)	0.024
LDL-cholesterol (per unit)	1.00 (0.99-1.00)	0.127	1.00 (0.99-1.00)	0.171		
Triglycerides (per unit)	1.00 (1.00-1.00)	0.017	1.00 (1.00-1.00)	0.001		
HbA1c (per unit)	1.04 (1.00-1.08)	0.046	1.06 (1.02-1.10)	0.003		
Hemoglobin (per unit)	0.94 (0.88-1.00)	0.043	1.01 (0.94-1.08)	0.834		
Statin treatment (yes versus no)	1.63 (1.30-2.02)	<0.0001	1.63 (1.30-2.04)	<0.0001	1.67 (1.33-2.08)	<0.0001
ACEI/ARB treatment (yes versus no)	1.87 (1.47-2.36)	<0.0001	1.67 (1.30-2.12)	<0.0001	1.68 (1.32-2.13)	<0.0001
Fibrate treatment (yes versus no)	1.13 (0.66-1.79)	0.645	1.43 (0.83-2.28)	0.186		

Multivariate model 1: Adjusted for age, disease duration, and baseline estimated glomerular filtration rate. Multivariate model 2: multivariate stepwise Cox regression analysis including all variables with a p-value <0.1 in model 1 listed in the table after adjustment for age, disease duration, and baseline estimated glomerular filtration rate. UACR, urinary albumin/creatinine ratio; HDL-C, high-density lipoprotein cholesterol; LDL, low-density lipoprotein; ACEI/ARB, angiotensin converting enzyme inhibitor or angiotensin receptor blocker.

Supplementary Table 4. Hazard ratios (HRs) of progression to moderately increased risk and very high risk stage of chronic kidney disease in female type 2 diabetic patients with a low risk stage of chronic kidney disease

	Univariate		Multivariate model 1		Multivariate model 2	
	HR (95% CI)	p-value	HR (95% CI)	p-value	HR (95% CI)	p-value
UACR (>8.15 versus ≤8.15 mg/g)	1.58 (1.32-1.91)	<0.0001	1.46 (1.21-1.77)	<0.0001	1.43 (1.17-1.75)	0.0004
Smoking (yes versus no)	0.99 (0.62-1.49)	0.958	1.21 (0.76-1.83)	0.406		
Body mass index (per unit)	1.04 (1.02-1.06)	<0.0001	1.05 (1.03-1.07)	<0.0001	1.03 (1.01-1.05)	0.003
Systolic blood pressure (per unit)	1.01 (1.00-1.01)	0.006	1.00 (0.99-1.01)	0.688		
Diastolic blood pressure (per unit)	1.01 (1.00-1.02)	0.021	1.01 (1.00-1.02)	0.021		
Total cholesterol (per unit)	1.00 (0.99-1.00)	0.120	1.00 (1.00-1.01)	0.012		
HDL-cholesterol (per unit)	0.99 (0.98-0.99)	0.040	0.99 (0.99-1.00)	0.114		
LDL-cholesterol (per unit)	0.99 (0.99-1.00)	0.467	1.00 (0.99-1.00)	0.877		
Triglycerides (per unit)	1.00 (1.00-1.00)	0.0001	1.00 (1.00-1.00)	<0.0001	1.00 (1.00-1.00)	0.003
HbA1c (per unit)	1.06 (1.02-1.10)	0.006	1.09 (1.04-1.13)	<0.0001	1.05 (1.01-1.10)	0.029
Hemoglobin (per unit)	0.90 (0.85-0.95)	0.001	0.93 (0.87-0.99)	0.032	0.90 (0.85-0.95)	0.0003
Statin treatment (yes versus no)	1.69 (1.37-2.07)	<0.0001	1.56 (1.25-1.93)	<0.0001	1.85 (1.47-2.32)	<0.0001
ACEI/ARB treatment (yes versus no)	1.81 (1.43-2.27)	<0.0001	1.67 (1.31-2.10)	<0.0001	1.58 (1.22-2.03)	0.001
Fibrate treatment (yes versus no)	2.05 (0.98-3.72)	0.057	2.36 (1.13-4.30)	0.025		

Multivariate model 1: Adjusted for age, disease duration, and baseline estimated glomerular filtration rate. Multivariate model 2: multivariate stepwise Cox regression analysis including all variables with a p-value <0.1 in model 1 listed in the table after adjustment for age, disease duration, and baseline estimated glomerular filtration rate. UACR, urinary albumin/creatinine ratio; HDL-C, high-density lipoprotein cholesterol; LDL, low-density lipoprotein; ACEI/ARB, angiotensin converting enzyme inhibitor or angiotensin receptor blocker.

Supplementary Table 5. Linear regression analysis of variables associated with urinary albumin/creatinine ratio levels

	Simple		Multiple	
Variable	β coefficient	p-value	β coefficient	p-value
Body mass index	0.085	<0.0001	-	-
Systolic blood pressure	0.155	<0.0001	0.156	<0.0001
Diastolic blood pressure	0.113	<0.0001	-	-
Smoking	0.004	0.785	-	-
Fasting glucose	0.055	<0.0001	-	-
HbA1c	0.080	<0.0001	0.117	<0.0001
Total cholesterol	0.053	<0.0001	-	-
Triglycerides	0.084	<0.0001	0.053	0.004
HDL-cholesterol	-0.070	<0.0001	-0.046	0.013
LDL-cholesterol	0.031	0.031	0.046	0.010
Uric acid	0.044	0.015	-	-
GPT	0.021	0.152	-	-
Creatinine	-0.008	0.579	-	-
eGFR	-0.035	0.015	-	-
Platelet	0.004	0.821	-	-
Hemoglobin	-0.046	0.006	-0.076	<0.0001
WBC count	0.031	0.063	-	-

In multiple linear stepwise regression analysis, all values were included for analysis. HDL, high-density lipoprotein; LDL, low-density lipoprotein; GPT, glutamic-pyruvic transaminase; eGFR, estimated glomerular filtration rate; WBC, white blood cell.
